# Efficacy and Safety of Guizhi Decoction AssociatedFormulas for Allergic Rhinitis: A Systematic Review

**DOI:** 10.1155/2021/3548740

**Published:** 2021-01-13

**Authors:** Shasha Yang, Qinwei Fu, Hua Deng, Jing Wu, Qinxiu Zhang, Limin Wang, Xianfeng Yao

**Affiliations:** ^1^The First Affiliated Hospital of Guizhou University of Traditional Chinese Medicine, Guiyang 550002, China; ^2^Hospital of Chengdu University of Traditional Chinese Medicine, Chengdu University of Traditional Chinese Medicine, Chengdu 610072, China; ^3^Guizhou University of Traditional Chinese Medicine, Guiyang 550025, China; ^4^School of Medical and Life Sciences/Reproductive & Women-Children Hospital, Chengdu University of Traditional Chinese Medicine, Chengdu 610041, China

## Abstract

In this study, we aim to evaluate the efficacy and safety of Guizhi Decoction associated formulas for the treatment of allergic rhinitis. A total of seven online databases were searched to collect studies published up to Feb 23^rd^, 2020. Study quality of each included article was evaluated by the Cochrane Collaboration risk of bias tool. Systematic reviews were conducted based on the Cochrane systematic review method by using RevMan 5.3 Software. Among the included trials, Guizhi Decoction associated formulas alone (or plus Western medicine, or acupoint-based therapy) were main therapies in experimental groups. Interventions in control groups include Western medicine, Guizhi Decoction associated formulas alone, Chinese patent medicine, and placebo control. Primary outcomes in this study include recovery rate, accumulative marked effective rate, accumulative effective rate, and recurrence rate. Finally, 23 trials involving 2281 participants were included. Results of systematic reviews show that Guizhi Decoction and associated formulas alone, plus Western medicine or plus acupoint-based therapies, were significantly better compared with using Western medicine alone in terms of efficacy. In addition, the formulas plus nasal TCM fumigation therapy could improve effective rate for AR treatment compared to using the formulas alone. More types and cases of adverse events were reported in the control groups (Western medicine alone), but events of included trials were all mild and did not need specific medical intervention. More RCTs of high quality, and large sample size, with appropriate blinding methods or nonblinded pragmatic trials of Guizhi Decoction and associated formulas for AR are needed.

## 1. Introduction

As one of the most common allergic diseases, allergic rhinitis (AR) can lead to symptoms including nasal sneezing, discharge, congestion, and itching, which may reduce patients' sleep quality, work productivity, and general functioning, thus often making them feel depressed and anxious [1, 2]. Patients with AR develop specific immunoglobulin *E* (IgE) antibody responses to indoor or outdoor environmental allergens, such as mites, pollen, house dust, animal dander, and mixed fungi, with exposure over time [3].

It was estimated that 20% to 40% of the population in the US and 10% to 20% of world population were suffering from AR [4, 5], with medical and hidden burden. Common therapies for AR include intranasal antihistamines, and novel methods of delivery for intranasal steroids [6]; however, some adverse events including minor nose bleeding and sedation or impairment of psychomotor function were reported [7, 8].

As a classic and important series of TCM decoctions, Guizhi Decoction and associated formulas have been used for over 1,800 years in China and are widely applied for AR till now. The efficacy of Guizhi Decoction and associated formulas matches the pathogenesis of AR according to TCM theory, and research showed that Guizhi Decoction associated formulas could inhibit cholinergic transdifferentiation of sympathetic nerves and improve the anatomical and functional denervation of sympathetic nerves [9]. However, the use of Guizhi Decoction and associated formulas in the treatment of AR beyond China is not popular, and the clinical efficacy and safety of Guizhi Decoction and associated formulas plus other treatments such as Western medicine and acupoint-based therapies were not certain.

Several reviews concerning TCM therapies especially herbal medicine decoctions for AR have been published [10, 11], while no study on efficacy and safety of Guizhi Decoction and associated formulas for AR has been conducted. The aim of this systematic review is to identify the clinical efficacy and safety of Guizhi Decoction associated formulas for the treatment of AR and to compare the efficacy and adverse effects in control groups by several comparisons.

## 2. Materials and Methods

### 2.1. Protocol and Registration

This systematic review was registered in PROSPERO, an international prospective register of systematic reviews, with the registration number CRD42020163034 (available from https://www.crd.york.ac.uk/prospero/display_record.php?RecordID=163034).

### 2.2. Search Strategy

We searched seven electronic databases, including Embase, PubMed, Cochrane Library, the China National Knowledge Infrastructure (CNKI), Technology Periodical Database (VIP), Wanfang Data Information Site, and SinoMed (CBM) up to Feb 23^rd^, 2020. The search strategy and inclusion criteria were decided according to the guidance of the PRISMA agreement [12]. We used the following two groups of search terms in English: (1) “allergic rhinitis”, “AR”, “anaphylactic rhinitis” connected with “OR”; (2) “Guizhi”, “Gui Zhi”, “*Cassia* twig”, “Ramulus Cinnamomi” connected with “OR”. The above search terms of (1) and (2) were connected with term “AND”. All searches were limited to trials of RCT in humans and were conducted in electronic databases by two authors independently. We also searched with related search terms in Chinese and searched the references of the original and review articles manually for possible related trials and also tried to get grey literatures identified through other sources. Detailed search strategies are in file S1.

### 2.3. Inclusion Criteria

In this systematic review, we searched and included trials according to the following criteria:

1. Trials including participants that were diagnosed with AR according to certain published guidelines with signs, symptoms, and laboratory findings.
2. Prospective randomized controlled trials (RCTs).
3. Trials in which Guizhi Decoction and associated formulas alone or plus other therapy(ies) were applied in experimental groups. The patients in control groups received conventional therapy(ies), other TCM therapies, or placebo regimens. Trials for more than one disease (AR) were excluded, such as asthma, cough, or conjunctivitis.
4. Efficacy was evaluated according to certain published guidelines. Primary outcomes included recovery rate, accumulative marked effective rate, accumulative effective rate, and recurrence rate. Accumulative effective rate is the sum of recovery rate (if reported), marked effective rate (if reported), and effective rate (if reported), and accumulative marked effective rate is the sum of the first and the second one.
5. Trials in Chinese or English.

### 2.4. Study Selection and Data Extraction

According to the above design, two reviewers (Qinwei Fu and Hua Deng) searched the online databases listed above and recorded the titles and abstracts of all the articles. Three evaluators (Limin Wang, Hua Deng, and Jing Wu) assessed the eligibility of these articles and made decisions on every research (inclusion or exclusion) independently. If they did not reach the same decision, the concerned articles were discussed with a fourth reviewer (Qinxiu Zhang). Three reviewers (Shasha Yang, Jing Wu, and Xianfeng Yao) extracted data independently from each study. Differences of extracted data were solved after discussion with a fourth reviewer (Qinxiu Zhang).

### 2.5. Quality Assessment

Quality assessment of all the trials included in this review was independently evaluated by three reviewers (Shasha Yang, Qinwei Fu, and Jing Wu) using the Cochrane Collaboration risk of bias tool by RevMan 5.3 Software. Any disagreement was resolved by discussions with a fourth reviewer (Qinxiu Zhang).

### 2.6. Statistical Analysis

This systematic review was performed with the RevMan 5.3. For outcome measures, the overall effect sizes were determined as the mean difference (MD) for continuous outcomes, and RR for binary outcomes with their 95% confidence intervals (CI), with *P* < 0.05  indicating significant differences for effect sizes. The *Q* and I2 test statistics were conducted to examine heterogeneity, with I2 > 50% indicating significant heterogeneity. Fixed-effects model was applied to statistical analysis. If the heterogeneity was still obvious (I2 > 50%) and more than six trials were included, then sensitivity analysis and subgroup analysis were conducted to identify certain variables or (and) trials leading to high heterogeneity. Exploration of publication bias by funnel plots was planned if more than six trials were included.

## 3. Results

### 3.1. Study Inclusion

Initially, 668 records were searched from seven databases with no grey literature reference. After the removal of duplicates, the records were decreased to 195. Based on titles and abstracts of records, we excluded 67 papers with reasons such as case reports, observational studies, uncontrolled studies, animal experiments, reviews, and studies with no randomization-control design and not related to Guizhi Decoction associated formulas for AR. The remaining 128 articles were downloaded for further selection, and 105 articles were excluded with reasons. Eventually, 23 trials of 22 studies (one three-arm study was recombined to two trials for comparison) were included [13–34] (Figure S1).

### 3.2. Study Characteristics

All 23 included RCTs were conducted in China and published in Chinese. In total, 2281 participants aging from 8 to 71 with AR from 50 days to 27 years were involved in 23 RCTs. Baseline characteristics were not detailed in several trials, but no significant difference among them between experimental and control groups (*P* < 0.05) was mentioned in all of the studies. As for the interventions of experimental groups, Guizhi Decoction associated formulas alone, combined with other TCM decoction/powder, with acupoint-based therapy (e.g., acupuncture, moxibustion, acupoint application, or auricular point pressing), with Western medicine (the same as the medicine used in the control groups mostly), or with Chinese patent medicine, were applied mainly. Although some of the specific prescriptions of Guizhi Decoction associated formulas in this study were different among the included trials, most of them have the effect of promoting *Qi* transmission and balancing *Yin* and *Yang* according to the theory of TCM and are widely used in the treatment of AR and many other diseases. As a result, they were considered as the same orientation. In addition, Western medicine alone was applied in control groups of 18 trials [13–16, 18, 19, 21–24, 26–28, 30–34], placebo control (normal saline, oral) in one trial [16], oral TCM decoction or powder, or Chinese patent medicine alone in two trials [16, 17], Chinese patent medicine combined with Western medicine in one trial [20], and Guizhi Decoction associated formulas plus TCM fumigation in one trial [29]. Detailed characteristics of the included trials are listed in Table S1.

### 3.3. Assessment of Quality and Bias

**Figure 1:**
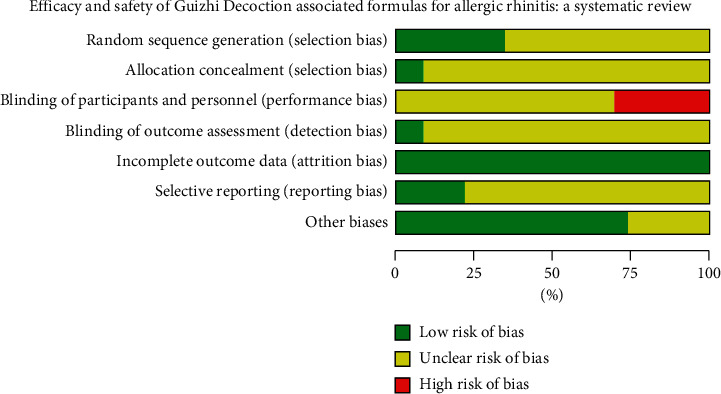
Risk of bias graph.

**Figure 2:**
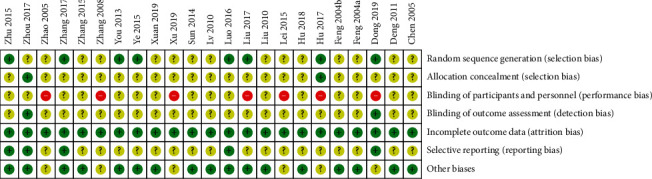
Risk of bias summary.

According to the results of Cochrane Collaboration risk of bias tool [35], the method of randomization was described clearly and appropriately in eight trials [15, 17, 21, 22, 27, 28, 31, 34] with no trial in high risk of bias. Two trials described the method of allocation concealment clearly [17, 33] while others were described unclearly. No trial reported blinding method in addition to two studies with specially assigned personnel in outcome assessment blinding [15, 33]. The bias for each trial is shown in Figure 1, and the bias summary is shown in Figure 2.

### 3.4. Efficacy of Guizhi Decoction Associated Formulas in AR Patients

#### 3.4.1. Guizhi Decoction Associated Formulas versus Western Medicine

Compared with the Western medicine groups, significant improvement was found in Guizhi Decoction associated formulas groups, including recovery rate without heterogeneity in five trials (RR = 1.67; P for RR < 0.01; 95% CI: 1.34–2.08; I^2^ = 0%) [13, 23, 25, 27, 28], accumulative marked effective rate without heterogeneity in nine trials (RR = 1.73; P for RR < 0.01; 95% CI: 1.47–2.02; I^2^ = 0%) [13, 16, 18, 23, 26–28, 30, 33], and accumulative effective rate with mild heterogeneity in nine trials (RR = 1.20; P for RR < 0.01; 95% CI: 1.13–1.27; I^2^ = 0%) [13, 16, 18, 23, 26–28, 30, 33] (Table 1, Figures S2 and S3).

In addition, the patients with Guizhi Decoction associated formulas were reported with significantly lower recurrence rate in four trials compared with the control groups after both three months (RR = 0.14; P for RR < 0.01; 95% CI: 0.04–0.47; I^2^ = 30%) and six months (RR = 0.20; P for RR < 0.01; 95% CI: 0.10–0.44; I^2^ = 34%) [13, 26–28]. Results of two trials also revealed that more cases with main symptoms disappeared in the experimental groups (RR = 1.95; P for RR = 0.05; 95% CI: 1.01–3.77; I^2^ = 79%) [27, 28]. Subgroup analysis was conducted, while no obvious difference was observed between the two trials. However, the duration of allergic rhinitis was not reported in one of them, which might be the course of considerable heterogeneity [28] (Table 1, Figure S4).

#### 3.4.2. Guizhi Decoction Associated Formulas plus Western Medicine versus Western Medicine

By comparison of the Guizhi Decoction associated formulas plus Western medicine groups versus Western medicine groups (Table 1, Figure S5), the pooled results favored the experimental groups on recovery rate without heterogeneity in two trials (RR = 1.26; P for RR = 0.13; 95% CI: 0.94–1.68; I^2^ = 0%) [14, 31], and on accumulative marked improvement rate (RR = 1.13; P for RR = 0.13; 95% CI: 0.96–1.33; I^2^ = 0%) and accumulative effective rate (RR = 1.22; P for RR < 0.01; 95% CI: 1.13–1.32; I^2^ = 0%) in four trials [14, 22, 31, 34].

#### 3.4.3. Guizhi Decoction Associated Formulas plus Acupoint-Based Therapy versus Western Medicine

Results of systematic review showed that Guizhi Decoction associated formulas plus certain acupoint-based therapy, including acupuncture, normal moxibustion, heat-sensitive moxibustion, and acupoint application, could provide better improvement on recovery rate (RR = 1.48; P for RR = 0.03; 95% CI: 1.03–2.12; I^2^: not applicable) [21], on accumulative marked improvement rate (RR = 1.07; P for RR = 0.89; 95% CI: 0.39–2.93; I^2^ = 76%) [15, 19], and on accumulative effective rate (RR = 1.13; P for RR = 0.18; 95% CI: 0.95–1.35; I^2^ = 66%) [15, 19, 21]. Subgroup analysis favored Guizhi Decoction associated formulas plus acupuncture with moxibustion compared with plus moxibustion or acupoint application on accumulative marked improvement rate and accumulative effective rate [15, 19, 21] (Table 1, Figure S6).

#### 3.4.4. Guizhi Decoction Associated Formulas versus Guizhi Decoction Associated Formulas plus TCM External Therapy

Control therapies of trials in this comparison include acupuncture, moxibustion, and local TCM fumigation. Compared with the experimental groups (Guizhi Decoction associated formulas), pooled results of two trials showed an increase in recovery rate (RR = 0.78; P for RR = 0.04; 95% CI: 0.61–0.99; I^2^ = 0%) and a significant improvement on accumulative effective rate (RR = 0.82; P for RR = 0.01; 95% CI: 0.71–0.96; I^2^ = 55%). In consideration of control therapies, subgroup analysis favored Guizhi Decoction associated formulas plus acupuncture with moxibustion compared with oral administration and nasal fumigation of Guizhi Decoction associated formulas on accumulative effective rate [17, 29] (Table 1, Figure S7).

### 3.5. Adverse Events Reported in Trials

Adverse events on the experimental groups were reported in four trials, and two trials reported no adverse event. For the experimental groups with Guizhi Decoction associated formulas applied, such events included palpitation (1 case), thirst (1 case), stomachache (1 case), and diarrhea (1 case) among the two studies covering 100 patients [33, 34]. Local burns recovered without scar after the Safflower Oil smeared (2 cases) and bucking (because of the smoke) relieved with a mask wore (1 case) were reported in one trial covering 30 patients, on whom heat-sensitive moxibustion plus Guizhi Decoction associated formulas were applied [15]. Liu and colleagues (Guizhi Decoction associated formulas plus acupoint application) mentioned adverse event as 5%, but with no details reported [21].

However, events were found in the control groups of all the six trials reported [15, 20, 21, 28, 33, 34], including drowsiness (13), fatigue (2), thirst (2), stomachache (3), and diarrhea (2) among the 100 patients in two trials [33, 34]. In addition, five cases (5 of 30) of slight adverse events (such as drowsiness, fatigue, and thirst) were reported in Dong 2019 [15], and Lin and colleagues found that nearly all the patients in control groups manifested fatigue, drowsiness, inattention, thirst, and weakness, with several cases of stomach discomfort [20]. Two trials mentioned adverse event as 4%, but also with no details reported [21, 28].

The adverse events reported in the experimental and control groups were all mild, and relieved or gone, or did not require specific intervention or medical evaluation. Events were not reported in the other 17 trials.

## 4. Discussion

To the best of our knowledge, this is the first systematic review of Guizhi Decoction associated formulas for AR. Guizhi Decoction is “the first and most applied decoction” in the Treatise on Febrile Disease, a TCM classic and regarded as one of the pioneers for TCM theory and practice. Originating from the late Eastern Han Dynasty in China about 1800 years ago, Guizhi Decoction and associated formulas are still widely used till now especially in China.

In TCM theory, AR falls into excess syndrome (stagnant heat of lung meridian, mainly) and deficiency syndrome (including lung *yang* deficiency, spleen *Qi* deficiency, and kidney *yang* deficiency, mainly) [36]. Guizhi Decoction is categorized as prescription for relieving exterior disorders in classification of prescriptions of TCM, while it is also widely recognized and applied in internal diseases through promoting *Qi* transmission and balancing *Yin* and *Yang*. Some formulas were transformed from Guizhi Decoction for specific purposes such as severe *Yang* deficiency, heat fire, or depression of exterior *Qi*, but their main principles are associated closely. Modern researches show that Guizhi Decoction associated formulas could inhibit cholinergic transdifferentiation of sympathetic nerves and improve the anatomical and functional denervation of sympathetic nerves [9]. Meanwhile, the formulas are also widely used in many diseases of internal, external, gynecologic, and other diseases such as colds, febrile diseases, various perspiration, digestive system diseases, respiratory diseases, ENT disease, throat disease, nervous system disease, cardiac autonomic neuropathy, and bone and joint diseases [37].

Results of our study show that, compared with applying Western medicine alone, higher total effective rate (including recovery rate, accumulative marked effective rate, and accumulative effective rate), lower recurrence rate (3 months and 6 months), and more cases with main symptoms of AR disappeared were reported in experimental groups, including applying Guizhi Decoction and associated formulas alone, plus Western medicine or plus acupoint-based therapies. In addition, there were fewer types and cases of adverse events in experimental groups. Some types of the events were found in both experimental and control groups, including thirst, stomachache, and diarrhea, while others appeared in Western medicine groups (drowsiness and fatigue) or experimental groups (local burns and bucking when applying moxibustion) only. However, adverse events reported were all mild and relieved or gone without specific medical intervention.

In addition, one comparison favored the control group (Guizhi Decoction and associated formulas plus TCM external therapy) with higher recovery rate and accumulative effective rate than experimental groups (Guizhi Decoction and associated formulas alone).

Acupoint-based therapies, such as acupuncture, moxibustion, acupoint catgut embedding, acupressure, and acupoint application, are important components of TCM external therapies. In China, some of the therapies such as acupuncture, moxibustion, acupoint catgut embedding, and acupoint application have been widely used in AR for symptoms relieving, severity reduction, adverse events decreasing, and life quality improving [38–41]. Such therapies in our study include heat-sensitive moxibustion, acupuncture, acupoint application, and auricular point pressing mainly, with TCM nasal fumigation in one study. It should be pointed out that some TCM therapies were effective for some allergic or respiratory diseases, including multiplex meridian interventions for asthma and Chinese herbal medicine for acute exacerbations of COPD, and for food allergy and eczema [42–44]. In addition, several studies of high quality in recent years approved that some acupoint-based therapies were not inferior to Western medicine in preventing and controlling some diseases, such as acupuncture and acupressure for cancer pain, acupuncture for chemotherapy-induced peripheral neuropathy symptoms, acupuncture for chronic stable angina, and acupressure combined with TCM footbath for diabetic peripheral neuropathy [45–48]. In addition, the therapies could reduce some medication intake, especially those that may have substantial addiction and adverse events.

As for study quality and risk of bias, all the 23 trials are RCTs, but placebo control was only employed in one study. Eight trials employed appropriate randomization method with clear statement, while it was in unclear risk of bias for the other 15 trials. Allocation concealment and blinding method were of unclear risk of bias in most trials. No study reported drop-out, and a protocol or registration ahead of experiment was only reported in two trials. As a result, double-blind, prospective, randomized, placebo-controlled trials of Guizhi Decoction and associated formulas as a treatment for AR are urgently need. In addition, concerning practical applicability and extrapolation in real-world situations, pragmatic trials without blinding have been suggested for achieving clinically relevant results in recent years [49, 50]. This type of trials is especially appropriate for TCM researches on efficacy and safety, considering the complexity and flexibility of TCM therapies.

## 5. Limitations

There are several limitations in our study. Firstly, trials included were of moderate to substantial risk of bias, such as lack of reporting about details of random sequence generation, concealment, and blinding of participants, personnel, and outcome assessment quality. This may lead to low quality of the included studies. Secondly, there was a wide range of publishing year (2004 to 2019) among the trials, which may lead to potential bias when they are pooled due to different editions of guidelines and standards (especially in diagnosis and outcomes). Finally, conforming to the criteria, assessment of publication bias was inapplicable for no more than 10 trials included in each comparison. The GRADE evidence profiles (EP) of our results are in Tables S2–S5, and most of them are in low or even very low quality.

As a result, more RCTs of high quality and large sample size, with appropriate blinding methods or nonblinded pragmatic trials by real-world researches, are needed to further improve and update our study.

## 6. Conclusion

In general, this systematic review demonstrated that applying Guizhi Decoction and associated formulas alone, plus Western medicine or plus acupoint-based therapies, may be safer and more effective for the treatment of AR than taking Western medicine alone. And the formulas plus nasal TCM fumigation could improve effective rate for AR treatment than using the formulas alone. More types and cases of adverse events were reported in the control groups (Western medicine alone), but events of included trials were all mild and did not need specific medical intervention. There is an urgent need for RCTs of high quality and large sample size, with appropriate blinding methods or nonblinded pragmatic trials of Guizhi Decoction and associated formulas for AR.

## Figures and Tables

**Table 1:** Summary of findings.

Intervention	Outcome	No. of trials	Participants (E: male/female)/(C: male/female)	Effect size (RR)	95% CI	*P* value Of effect size	I^2^ value
GZDAF vs. WM	Recovery rate	5	(154/109)/(138/99)	1.67	1.34 to 2.08	<0.01	0%
	Accumulative marked effective rate	9	(269/192)/(247/183)	1.73	1.47 to 2.02		0%
	Accumulative marked rate			1.20	1.13 to 1.27		28%
	Recurrence rate (3 months)	4	(125/110)/(83/72)	0.14	0.04 to 0.47		30%
	Recurrence rate (6 months)			0.20	0.10 to 0.44		34%
	Cases of main symptoms disappearance (subgroup 1)	1	(18/14)/(20/12)	1.44	1.02 to 2.05	0.05	NA
	Cases of main symptoms disappearance (subgroup 2)	1	(21/19)/(25/15)	2.75	1.68 to 4.51	<0.01	NA

GZDAF + WM vs. WM	Recovery rate	2	A∗	1.26	0.94 to 1.68	0.13	0%
	Accumulative marked effective rate	4	B∗	1.13	0.96 to 1.33		
	Accumulative marked rate			1.22	1.13 to 1.32	<0.01	

GZDAF + ABT vs. WM	Recovery rate	1	(52/48)/(27/23)	1.48	1.03 to 2.12	0.03	NA
	Accumulative marked effective rate (subgroup 1)	1	(15/15)/(14/16)	0.6	0.25 to 1.44	0.25	NA
	Accumulative marked effective rate (subgroup 2)	1	(20/20)/(21/19)	1.67	1.05 to 2.66	0.03	NA
	Accumulative marked rate (subgroup 1)	2	(35/35)/(35/35)	1.05	0.94 to 1.17	0.38	0%
	Accumulative marked rate (subgroup 2)	1	(20/20)/(21/19)	1.41	1.12 to 1.77	<0.01	NA

GZDAF vs. GZDAF + TCMET	Recovery rate	2	(67/95)/(60/82)	0.78	0.61 to 0.99	0.04	0%
	Accumulative marked rate (subgroup 1)	1	(43/77)/(38/62)	0.77	0.66 to 0.89	<0.01	NA
	Accumulative marked rate (subgroup 2)	1	(24/18)/(22/20)	0.88	0.77 to 1	0.05	NA

E: experimental group; C: control group; GZDAF: Guizhi Decoction associated formulas; WM: Western medicine; ABT: acupoint-based therapy; TCMET: TCM external therapy; NA: not applicable; A∗: 61 for male (E and C)/53 for female (E and C) in Deng 2011, and (33/34)/(31/26) in Zhang 2017; B∗: 61 for male (E and C)/53 for female (E and C) in Deng 2011, 72 for male (E and C)/62 for female (E and C) in Luo 2016, and (59/38)/(54/53) in Zhang 2017 + Zhu 2015.

## Data Availability

The data used in the study are available upon request to the corresponding author.

## Abbreviations:

TCM: Traditional Chinese medicine
RR: Risk ratio
MD: Mean difference
CI: Confidence interval.

## Supplementary Materials

Supplementary Materials Detailed search strategies, characteristics of included trials, GRADE evidence profiles (EP) of our results, the flow diagram of the study selection process, and forest plots of the comparisons in our study are listed in supplementary materials document.
