# Supplementary material for: Efficacy and Safety of Guizhi Decoction AssociatedFormulas for Allergic Rhinitis: A Systematic Review
Source: Evid Based Complement Alternat Med. 2021 Jan 13;2021:3548740. doi: 10.1155/2021/3548740 (PMC7817259; doi:10.1155/2021/3548740)
Supplement: Supplementary Materials — Detailed search strategies, characteristics of included trials, GRADE evidence profiles (EP) of our results, the flow diagram of the study selection process, and forest plots of the comparisons in our study are listed in supplementary materials document. [file 3548740.f1.docx]

[file S1: Detailed search strategies: 2](#_Toc60261836)

[table S1: Detailed characteristics of included trials 5](#_Toc60261837)

[table S2: GRADE evidence profiles (EP) of Guizhi Decoction associated formulas versus Western medicine 8](#_Toc60261838)

[table S3: GRADE evidence profiles (EP) of Guizhi Decoction associated formulas plus Western medicine versus Western medicine 11](#_Toc60261839)

[table S4: GRADE evidence profiles (EP) of Guizhi Decoction associated formulas plus acupoint-based therapy versus Western medicine 13](#_Toc60261840)

[table S5: GRADE evidence profiles (EP) of Guizhi Decoction associated formulas versus Guizhi Decoction associated formulas plus TCM external therapy 15](#_Toc60261841)

[figure S1: The flow diagram of the study selection process 17](#_Toc60261842)

[figure S2: Guizhi Decoction associated formulas versus Western medicine 18](#_Toc60261843)

[Figure S3: Guizhi Decoction associated formulas versus Western medicine 19](#_Toc60261844)

[Figure S4: Guizhi Decoction associated formulas versus Western medicine-continued 20](#_Toc60261845)

[figure S5: Guizhi Decoction associated formulas plus Western medicine versus Western medicine 21](#_Toc60261846)

[Figure S6: Guizhi Decoction associated formulas plus acupoint-based therapy versus Western medicine 22](#_Toc60261847)

[figure S7: Guizhi Decoction associated formulas versus Guizhi Decoction associated formulas plus TCM external therapy 23](#_Toc60261848)

file S1: Detailed search strategies:

(1) Search strategy for Pubmed:

(allergic rhinitis[Title/Abstract] OR rhinallergosis[Title/Abstract] OR nasal allergy[Title/Abstract] OR AR[Title/Abstract] OR anaphylactic rhinitis[Title/Abstract]) AND (Guizhi decoction[Title/Abstract] OR Guizhi Tang[Title/Abstract] OR ramuli cinnamomi decoction[Title/Abstract] OR Cinnamon Twig Decoction[Title/Abstract] OR Guizhi[Title/Abstract] OR ramuli cinnamomi[Title/Abstract] OR Cinnamon Twig Decoction[Title/Abstract])

(2) Search strategy for Embase:

('allergic rhinitis':ab OR rhinallergosis:ab OR 'nasal allergy':ab OR AR:ab OR 'anaphylactic rhinitis':ab) AND ('Guizhi decoction':ab OR 'Guizhi Tang':ab OR 'ramuli cinnamomi decoction':ab OR 'Cinnamon Twig Decoction':ab OR Guizhi:ab OR 'ramuli cinnamomi':ab OR 'Cinnamon Twig Decoction':ab)

(3) Search strategy for Cochrane Library

#1 allergic rhinitis[Title Abstract Keyword]

#2 rhinallergosis

#3 nasal allergy

#4 AR

#5 anaphylactic rhinitis

#6 #1 OR #2 OR #3 OR #4 OR #5

#7 Guizhi decoction

#8 Guizhi Tang

#9 ramuli cinnamomi decoction

#10 Cinnamon Twig Decoction

#11 Guizhi

#12 ramuli cinnamomic

#13 Cinnamon Twig Decoction

#14 #7 OR #8 OR #9 OR #10 OR #11 OR #12 OR #13

#15 #6 AND #14

(4) Search strategy for the China National Knowledge Infrastructure (CNKI):

(SU='Bian ying xing bi yan' OR SU=' Guo min xing bi yan' OR SU='Qiu ti' OR SU='Bi qiu' OR SU='Hua fen zheng') AND (SU='Gui zhi tang' OR SU='Tiao he yin wei' OR SU=' Gui zhi ')

(5) Search strategy for the Wanfang Data Information Site:

Subject:(" Bian ying xing bi yan" or " Guo min xing bi yan" or " Qiu ti " or " Bi qiu " or " Hua fen zheng ") and Subject:(" Gui zhi tang " or " Tiao he yin wei " or " Gui zhi ")

(6) Search strategy for the Technology Periodical Database (VIP) and the SinoMed (CBM):

(M=( Bian ying xing bi yan OR Guo min xing bi yan OR Qiu ti OR Bi qiu OR Hua fen zheng)) AND (M=( Gui zhi tang OR Tiao he yin wei OR Gui zhi))

table S1: Detailed characteristics of included trials

| **Study** | **Age (E/C)** | **Duration (E/C)** | **Male (E/C)** | **Female (E/C)** | **Interventions (E/C)** | **Period of treatment (E/C)** | **Outcomes** | **Advert events (E/C)** |
| --- | --- | --- | --- | --- | --- | --- | --- | --- |
| Chen 2005 | 10-65 (41.60±4.36)/11-65 (39.45±7.12) Y | 1-20/1-18 Y | 35/16 | 21/14 | GZD (qd) + YPFP (qd)/ Terfenadine tablet (qd) | 1M | 1. Recovery rate; 2. Accumulative marked effective rate; 3. Accumulative effective rate; 4. Recurrence rate (3M/6M/1Y). | NR |
| Deng 2011 | 16-52 (31.6±9.78) Y | NR | 61 | 53 | CHGZD (bid) + Levocetirizine tablets (bid)/ Levocetirizine tablets (bid) | 20D | 1. Recovery rate; 2. Accumulative marked effective rate; 3. Accumulative effective rate; 4. Symptom score. | NR |
| Dong 2019 | 18-59 (33.13±8.51)/18-50 (35.33±9.86) Y | 0.5-40/0.5-20 Y | 15/14 | 15/16 | Heat-sensitive moxibustion (NR) + GZD (bid)/ Fluticasone propionate nasal spray (qd-bid) + Loratadine tablet (qd) | 20D | 1. Recovery rate (with follow-up for 2W); 2. Accumulative effective rate (with follow-up for 2W); 3. Nasal symptom and sign score; 4. RQLQ score. | Y/ Y |
| Feng 2004 a | 18-64 (37.74±14.57)/18-65 (38.17±14.21) Y | 0.6-11 (6.1±4.3)/1-27 (11.31±8.17) Y | 22/22 | 21/21 | Allergic rhinitis oral liquid (tid)/ Normal saline (oral administration) (tid) | 4W | 1. Accumulative marked effective rate; 2. Accumulative effective rate; 3. Serum IL-4; 4. Serum IgE. | NR |
| Feng 2004 b | 18-64 (37.74±14.57)/18-65 (39.87±14.36) | 0.6-11 (6.1±4.3)/0.6-10 (5.4±3.3) Y | 22/20 | 21/23 | Allergic rhinitis oral liquid (tid)/ Biyankang tablet (tid) | 4W | 1. Accumulative marked effective rate; 2. Accumulative effective rate; 3. Serum IL-4; 4. Serum IgE. | NR |
| Hu 2017 | 9-65 (37.6±9.9)/10-66 (38.8±9.1) Y | 0.6-11 (6.1±4.3)/0.6-10 (5.4±3.3) Y | 24/22 | 18/20 | GZPFD (qd) / GZPFD (qd) + Acupuncture (NR) + Moxibustion (NR) | 1W | 1. Recovery rate; 2. Accumulative effective rate; 3. Advert event rate. | NR |
| Hu 2018 | 39.20±11.71/38.10±12.22 Y | NR | 12/13 | 18/17 | HQGZWWD (bid) + CEZP (bid)/ Budesonide nasal spray (bid) | 30D | 1. Accumulative marked effective rate; 2. Accumulative effective rate; 3. Total score of TCM symptoms; 4. CD4+, CD25+ and Treg (Peripheral blood). | NR |
| Lei 2015 | 9-65 (36.1±15.1)/10-68 (37.0±15.2) Y | NR | 20/21 | 20/19 | GZPFD (tid) + Acupuncture (tid) + Moxibustion (tid)/ Ordinary Western medicine | NR | 1. Accumulative marked effective rate; 2. Accumulative effective rate. | NR |
| Lin 2010 | 18-45 (mean=29.1)/18-47 (men=30.4) Y | 3 (M)-11 (2.5)/4 (M)-12 (3.1) Y | 13/15 | 17/15 | XQGZD (qd) + Nuckear dairy injection (biw)/ Biyankang tablet (tid) + Nuckear dairy injection (biw) | 14D | 1. Recovery rate; 2. Accumulative effective rate. | N/ Y |
| Liu 2017 | 18-60 (41.0±8.9)/18-59 (42.0±9.6) Y | 2-15 (8.8±2.9)/2-14 (8.3±3.2) Y | 52/27 | 48/23 | GZD + CEZP + Acupoint application (1 per 3D) / Loratadine dispersible tablets (qd)+ Azostyne nasal spray (bid) | 2W | 1. Recovery rate; 2. Accumulative effective rate; 3. Advert event rate. | Y/ Y |
| Luo 2016 | 18-60 (31.5±5.4) Y | 1-5 (2.1±0.7) Y | 72 | 62 | GZHQD (qd) + Levocetirizine hydrochloride tablets (qd) + Budesonide nasal spray (qd)/ Levocetirizine hydrochloride tablets (qd) + Budesonide nasal spray (qd) | 60D | 1. Accumulative marked effective rate; 2. Accumulative effective rate; 3. T lymphocyte subsets (Peripheral blood); 4. IgA; 5. IgM; 6. IgG. | NR |
| Lv 2010 | 15-53 (mean=35)/14-55 (men=34) Y | 50 (D)-15 (mean=4.9)/2 (M)-16 (men=4.7) Y | 29/28 | 26/27 | GZLGMLD (qd)/ Loratadine tablet (qd) | 30D | 1. Recovery rate; 2. Accumulative marked effective rate; 3. Accumulative effective rate. | NR |
| Sun 2014 | 18-52 (37.13±11.11) Y | 1-12 (7.33±3.56) Y | 33 | 31 | GZD (bid)/ Desloratadine citrate disodium tablets (qd) | 14D | 1. Symptom score; 2. RQLQ score. | NR |
| Xu 2019 | 29-58 (37.28±16.38)/26-63 (36.91±17.05) Y | 8.35±1.47/8.41±1.50 Y | 17/15 | 13/15 | Levocetirizine hydrochloride dispersible tablets (qd) +GZD (bid) + Auricular point pressing (5-7/D) /Levocetirizine hydrochloride dispersible tablets (qd) | NR | 1. Recovery rate; 2. Accumulative marked effective rate; 3. Accumulative effective rate; 4. TCM symptom score. | NR |
| Xuan 2019 | 19-64 (34.05±9.84)/20-62 (33.87±10.05) Y | 1-5 (2.97±1.06)/1-4 (2.34±0.95) Y | 51/49 | 29/31 | GZD (qd) + MHFZXXD (qd)/ Loratadine tablet(bid) | 4W | 1. Recovery rate; 2. Accumulative effective rate; 3. TCM symptom score; 4. Recurrence rate (3M/6M). | NR |
| Ye 2015 | 18-65 (32.50±10.12)/18-65 (34.66±12.63) Y | 9-48 (27.86±9.30)/12-56 (29.73±10.56) M | 18/20 | 14/12 | GZD (bid) + MHFZXXD (bid) / Loratadine tablet (qd) + Transfer factor capsule (tid) | 28D | 1. Recovery rate; 2. Accumulative marked effective rate; 3. Accumulative effective rate; 4. Cases of disappearance of main symptoms; 5. Recurrence rate (3M/6M). | NR |
| You 2013 | 10-60/12-58 Y | NR | 21/25 | 19/15 | GZD (qd) + XSP (qd)/ Loratadine tablet (qd) + Transfer factor capsule (tid) | 28D | 1. Recovery rate; 2. Accumulative marked effective rate; 3. Accumulative effective rate; 4. Cases of disappearance of main symptoms; 5. Recurrence rate (3M/6M); 6. Advert event rate. | N/ Y |
| Zhang 2008 | 13-70 (mean=28)/5-70 (men=30) Y | 3-17 (mean=4.3)/2-16 (men=3.9) Y | 43/38 | 77/62 | YPFP + GZD + CEZP (oral administration only) (qd)/YPFP + GZD + CEZP (oral administration and nasal fumigation) (qd) | 45D | 1. Recovery rate; 2. Accumulative effective rate. | NR |
| Zhang 2015 | 17-49 (mean=36)/18-48 (men=38) Y | 2-7/2-6 Y | 43/40 | 22/20 | YPFP (bid) + GZD (bid)/ Terfenadine tablet (bid) | 20D | 1. Accumulative marked effective rate; 2. Accumulative effective rate. | NR |
| Zhang 2017 | 34.1±3.9/33.7±4.1 Y | 3.6±0.7/3.4±0.9 Y | 33/31 | 24/26 | CHGZD (bid) + Cetirizine hydrochloride tablets (qd)/ Cetirizine hydrochloride tablets(qd) | 3M | 1. Recovery rate; 2. Accumulative marked effective rate; 3. Accumulative effective rate; 4. Clinical symptom disappearance time; 5. Score of symptoms and signs; 6. Inflammatory cytokine level. | NR |
| Zhao 2005 | 8-43 (mean=21.7) Y | A* | 50 | 34 | GZPFD (qd) + Acupuncture (qd) + Moxibustion (qd)/ Ordinary Western medicine* | NR | Scores of symptoms and signs. | NR |
| Zhou 2017 | 15-65 (28.5±7.0)/15-64 (30.0±5.4) Y | 1-28/10 (M)-29 Y | 38/36 | 22/24 | HQGZWWD (tib)/ Loratadine tablet (qd) | 30D | 1. Accumulative marked effective rate; 2. Accumulative effective rate; 3. Total score of symptoms and signs; 4.IL-6; 5. IL-8. | Y/ Y |
| Zhu 2015 | 13-68 (41.2±3.6)/21-71 (42.6±4.2) Y | 3(W)-10 (6.2±2.8)/4(W)-11 (6.8±2.5) Y | 26/23 | 14/17 | CHGZD (bid) + Cetirizine tablets (qd)/ Cetirizine tablets (qd) | 20D | 1. Accumulative marked effective rate; 2. Accumulative effective rate; 3. IgE; 4. Symptom score. | Y/ Y |
| Y: Yeay; M: Month; W: Week; D: Day; A*: 6M-1Y: 20, 1Y-2Y: 49, >2Y: 15; NR: Not reported; N: None; Y: Yes; GZD: Guizhi Decoction; YPFP: Yu-Ping-Feng Powder; CHGZD: Chaihu Guizhi Decoction; GZPFD: Guizhi-Ping-Feng Decoction; HQGZWWD: Huangqi Guizhi Wuwu Decoction; CEZP: Cang-Er-Zi Powder; XQGZD: Xinqi Guizhi Decoction; Guizhi and Longgu Muli Decoction: GZLGMLD; Mahuang Fuzi Xixin Decoction: MHFZXXD; Xingsu Powder: XSP; Ordinary Western medicine*: such as antihistamines and glucocorticoids; RQLQ: Rhinoconjunctivitis Quality of Life Questionnaire. | | | | | | | | |

table S2: GRADE evidence profiles (EP) of Guizhi Decoction associated formulas versus Western medicine

| **Certainty assessment** | | | | | | | **No. of patients** | | **Effect** | | **Certainty** | **Importance** |
| --- | --- | --- | --- | --- | --- | --- | --- | --- | --- | --- | --- | --- |
| **No. of studies** | **Design** | **Risk of bias** | **Inconsistency** | **Indirectness** | **Imprecision** | **Other considerations** | **GZDAF** | **WM** | **Relative (95% CI)** | **Absolute (95% CI)** |  |  |
| **A1 - Recovery rate** | | | | | | | | | | | | |
| 5 | RCT | Serious | Not serious | Serious | Not serious | None | 137/263 (52.1%) | 71/237 (30.0%) | **RR 1.67** (1.34 to 2.08) | **201 more per 1,000** (from 102 more to 324 more) | ⨁⨁◯◯ LOW | N/A |
| **A1 - Accumulative marked improvement rate** | | | | | | | | | | | | |
| 9 | RCT | Serious | Not serious | Serious | Not serious | Publication bias | 254/461 (55.1%) | 135/430 (31.4%) | **RR 1.73** (1.47 to 2.02) | **229 more per 1,000** (from 148 more to 320 more) | ⨁◯◯◯ VERY LOW | N/A |
| **A1 - Accumulative effective rate** | | | | | | | | | | | | |
| 9 | RCT | Serious | Not serious | Serious | Not serious | None | 424/461 (92.0%) | 329/430 (76.5%) | **RR 1.20** (1.13 to 1.27) | **153 more per 1,000** (from 99 more to 207 more) | ⨁⨁◯◯  LOW | N/A |
| **A2 A3 A4 - Recurrence rate (3 months)** | | | | | | | | | | | | |
| 4 | RCT | Serious | Not serious | Serious | Not serious | None | 4/180 (2.2%) | 39/137 (28.5%) | **RR 0.14** (0.04 to 0.47) | **245 fewer per 1,000** (from 273 fewer to 151 fewer) | ⨁⨁◯◯  LOW | N/A |
| **A2 A3 A4 - Recurrence rate (6 months)** | | | | | | | | | | | | |
| 4 | RCT | Serious | Not serious | Serious | Not serious | None | 12/180 (6.7%) | 57/137 (41.6%) | **RR 0.20** (0.10 to 0.44) | **333 fewer per 1,000** (from 374 fewer to 233 fewer) | ⨁⨁◯◯  LOW | N/A |
| **A2 A3 A4 - Cases with main symptoms disappeared (subgroup 1)** | | | | | | | | | | | | |
| 1 | RCT | Serious | Serious | Not serious | Serious | None | 26/32 (81.3%) | 18/32 (56.3%) | **RR 1.44** (1.02 to 2.05) | **247 more per 1,000** (from 11 more to 591 more) | ⨁◯◯◯ VERY LOW | N/A |
| **A2 A3 A4 - Cases with main symptoms disappeared (subgroup 2)** | | | | | | | | | | | | |
| 1 | RCT | Serious | Serious | Not serious | Serious | None | 33/40 (82.5%) | 12/40 (30.0%) | **RR 2.75** (1.68 to 4.51) | **525 more per 1,000** (from 204 more to 1,000 more) | ⨁◯◯◯ VERY LOW | N/A |
| **CI:** Confidence interval; **RR:** Risk ratio; **GZDAF:** Guizhi Decoction associated formulas; **WM:** Western medicine. | | | | | | | | | | | | |

table S3: GRADE evidence profiles (EP) of Guizhi Decoction associated formulas plus Western medicine versus Western medicine

| **Certainty assessment** | | | | | | | **No. of patients** | | **Effect** | | **Certainty** | **Importance** |
| --- | --- | --- | --- | --- | --- | --- | --- | --- | --- | --- | --- | --- |
| **No. of studies** | **Design** | **Risk of bias** | **Inconsistency** | **Indirectness** | **Imprecision** | **Other considerations** | **GZDAF+WM** | **WM** | **Relative (95% CI)** | **Absolute (95% CI)** |  |  |
| **B1 - Recovery rate** | | | | | | | | | | | | |
| 2 | RCT | Serious | Not serious | Not serious | Not serious | None | 59/124 (47.6%) | 47/124 (37.9%) | **RR 1.26** (0.94 to 1.68) | **99 more per 1,000** (from 23 fewer to 258 more) | ⨁⨁⨁◯ MODERATE | N/A |
| **B1 - Accumulative marked improvement rate** | | | | | | | | | | | | |
| 4 | RCT | Serious | Not serious | Serious | Not serious | None | 136/240 (56.7%) | 120/240 (50.0%) | **RR 1.13** (0.96 to 1.33) | **65 more per 1,000** (from 20 fewer to 165 more) | ⨁⨁◯◯  LOW | N/A |
| **B1 - Accumulative effective rate** | | | | | | | | | | | | |
| 4 | RCT | Serious | Not serious | Serious | Not serious | None | 224/240 (93.3%) | 184/240 (76.7%) | **RR 1.22** (1.13 to 1.32) | **169 more per 1,000** (from 100 more to 245 more) | ⨁⨁◯◯  LOW | N/A |
| **CI:** Confidence interval; **RR:** Risk ratio; **GZDAF:** Guizhi Decoction associated formulas; **WM:** Western medicine. | | | | | | | | | | | | |

table S4: GRADE evidence profiles (EP) of Guizhi Decoction associated formulas plus acupoint-based therapy versus Western medicine

| **Certainty assessment** | | | | | | | **No. of patients** | | **Effect** | | **Certainty** | **Importance** |
| --- | --- | --- | --- | --- | --- | --- | --- | --- | --- | --- | --- | --- |
| **No. of studies** | **Design** | **Risk of bias** | **Inconsistency** | **Indirectness** | **Imprecision** | **Other considerations** | **GZDAF+ABT** | **WM** | **Relative (95% CI)** | **Absolute (95% CI)** |  |  |
| **C1 - Recovery rate** | | | | | | | | | | | | |
| 1 | RCT | Very serious | Serious | Not serious | Serious | None | 62/100 (62.0%) | 21/50 (42.0%) | **RR 1.48** (1.03 to 2.12) | **202 more per 1,000** (from 13 more to 470 more) | ⨁◯◯◯ VERY LOW | N/A |
| **C1 - Accumulative marked improvement (subgroup 1)** | | | | | | | | | | | | |
| 1 | RCT | Very serious | Serious | Serious | Serious | None | 6/30 (20.0%) | 10/30 (33.3%) | **RR 0.60** (0.25 to 1.44) | **133 fewer per 1,000** (from 250 fewer to 147 more) | ⨁◯◯◯ VERY LOW | N/A |
| **C1 - Accumulative marked improvement (subgroup 2)** | | | | | | | | | | | | |
| 1 | RCT | Very serious | Serious | Not serious | Serious | None | 25/40 (62.5%) | 15/40 (37.5%) | **RR 1.67** (1.05 to 2.66) | **251 more per 1,000** (from 19 more to 623 more) | ⨁◯◯◯ VERY LOW | N/A |
| **C1 - Accumulative effective rate (subgroup 1)** | | | | | | | | | | | | |
| 2 | RCT | Very serious | Not serious | Serious | Not serious | None | 118/130 (90.8%) | 69/80 (86.3%) | **RR 1.05** (0.94 to 1.17) | **43 more per 1,000** (from 52 fewer to 147 more) | ⨁◯◯◯ VERY LOW | N/A |
| **C1 - Accumulative effective rate (subgroup 2)** | | | | | | | | | | | | |
| 1 | RCT | Very serious | Serious | Not serious | Serious | None | 38/40 (95.0%) | 27/40 (67.5%) | **RR 1.41** (1.12 to 1.77) | **277 more per 1,000** (from 81 more to 520 more) | ⨁◯◯◯ VERY LOW | N/A |
| **CI:** Confidence interval; **RR:** Risk ratio; **GZDAF:** Guizhi Decoction associated formulas; **ABT:** acupoint-based therapy; **WM:** Western medicine. | | | | | | | | | | | | |

table S5: GRADE evidence profiles (EP) of Guizhi Decoction associated formulas versus Guizhi Decoction associated formulas plus TCM external therapy

| **Certainty assessment** | | | | | | | **No. of patients** | | **Effect** | | **Certainty** | **Importance** |
| --- | --- | --- | --- | --- | --- | --- | --- | --- | --- | --- | --- | --- |
| **No. of studies** | **Design** | **Risk of bias** | **Inconsistency** | **Indirectness** | **Imprecision** | **Other considerations** | **GZDAF** | **GZDAF+TCMET** | **Relative (95% CI)** | **Absolute (95% CI)** |  |  |
| **C1 - Recovery rate** | | | | | | | | | | | | |
| 1 | RCT | Very serious | Serious | Not serious | Serious | None | 62/100 (62.0%) | 21/50 (42.0%) | **RR 1.48** (1.03 to 2.12) | **202 more per 1,000** (from 13 more to 470 more) | ⨁◯◯◯ VERY LOW | N/A |
| **C1 - Accumulative marked improvement (subgroup 1)** | | | | | | | | | | | | |
| 1 | RCT | Very serious | Serious | Serious | Serious | None | 6/30 (20.0%) | 10/30 (33.3%) | **RR 0.60** (0.25 to 1.44) | **133 fewer per 1,000** (from 250 fewer to 147 more) | ⨁◯◯◯ VERY LOW | N/A |
| **C1 - Accumulative marked improvement (subgroup 2)** | | | | | | | | | | | | |
| 1 | RCT | Very serious | Serious | Not serious | Serious | None | 25/40 (62.5%) | 15/40 (37.5%) | **RR 1.67** (1.05 to 2.66) | **251 more per 1,000** (from 19 more to 623 more) | ⨁◯◯◯ VERY LOW | N/A |
| **C1 - Accumulative effective rate (subgroup 1)** | | | | | | | | | | | | |
| 2 | RCT | Very serious | Not serious | Serious | Not serious | None | 118/130 (90.8%) | 69/80 (86.3%) | **RR 1.05** (0.94 to 1.17) | **43 more per 1,000** (from 52 fewer to 147 more) | ⨁◯◯◯ VERY LOW | N/A |
| **C1 - Accumulative effective rate (subgroup 2)** | | | | | | | | | | | | |
| 1 | RCT | Very serious | Serious | Not serious | Serious | None | 38/40 (95.0%) | 27/40 (67.5%) | **RR 1.41** (1.12 to 1.77) | **277 more per 1,000** (from 81 more to 520 more) | ⨁◯◯◯ VERY LOW | N/A |
| **CI:** Confidence interval; **RR:** Risk ratio; **GZDAF:** Guizhi Decoction associated formulas; **TCMET:** TCM external therapy. | | | | | | | | | | | | |


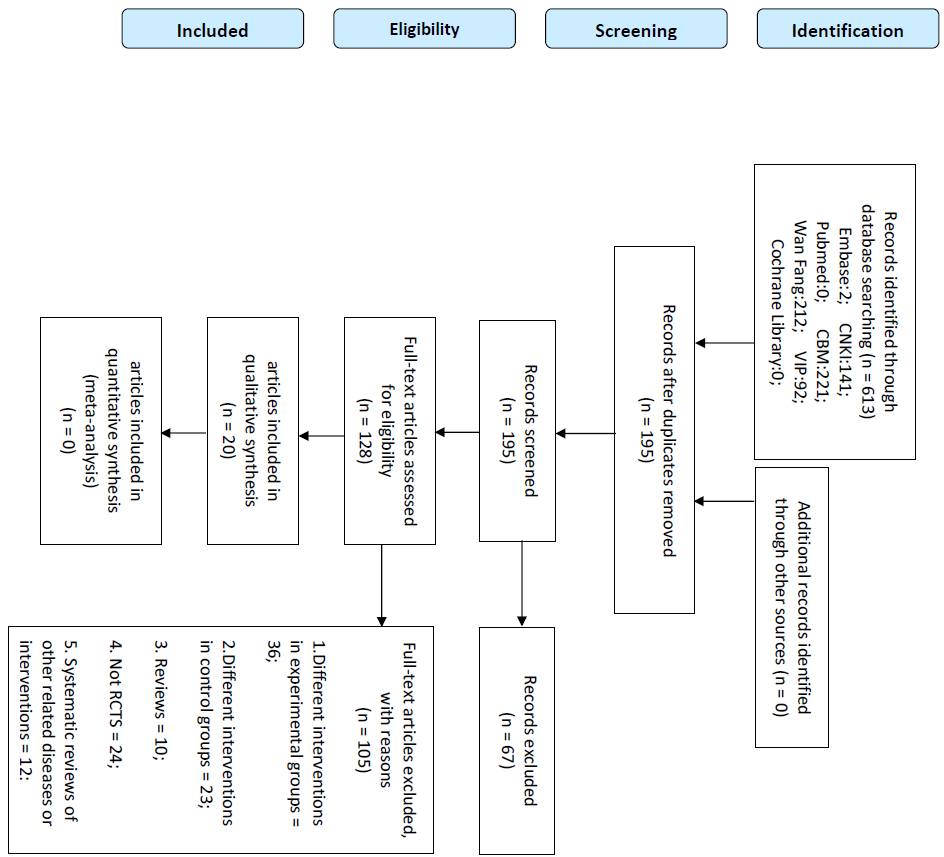


figure S1: The flow diagram of the study selection process


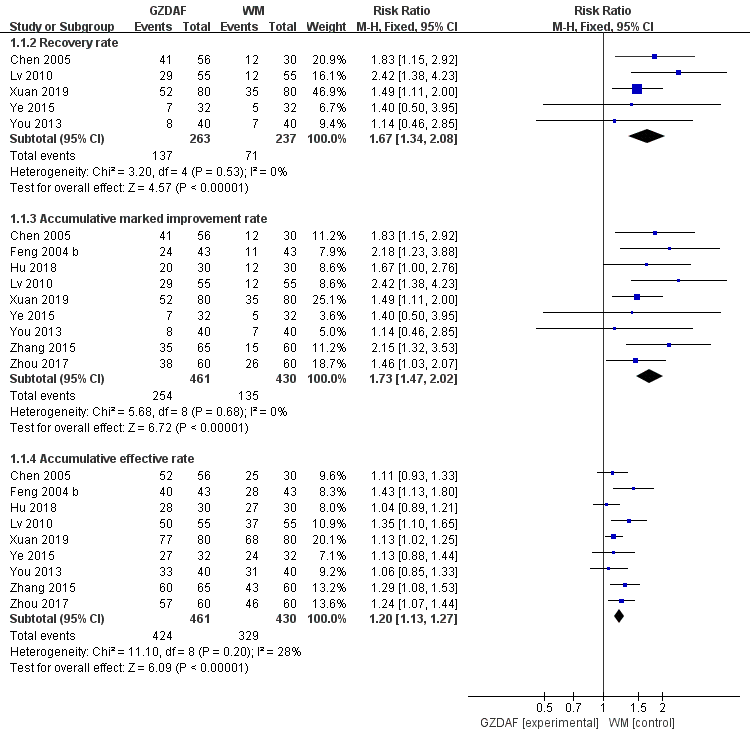


figure S2: Guizhi Decoction associated formulas versus Western medicine

*
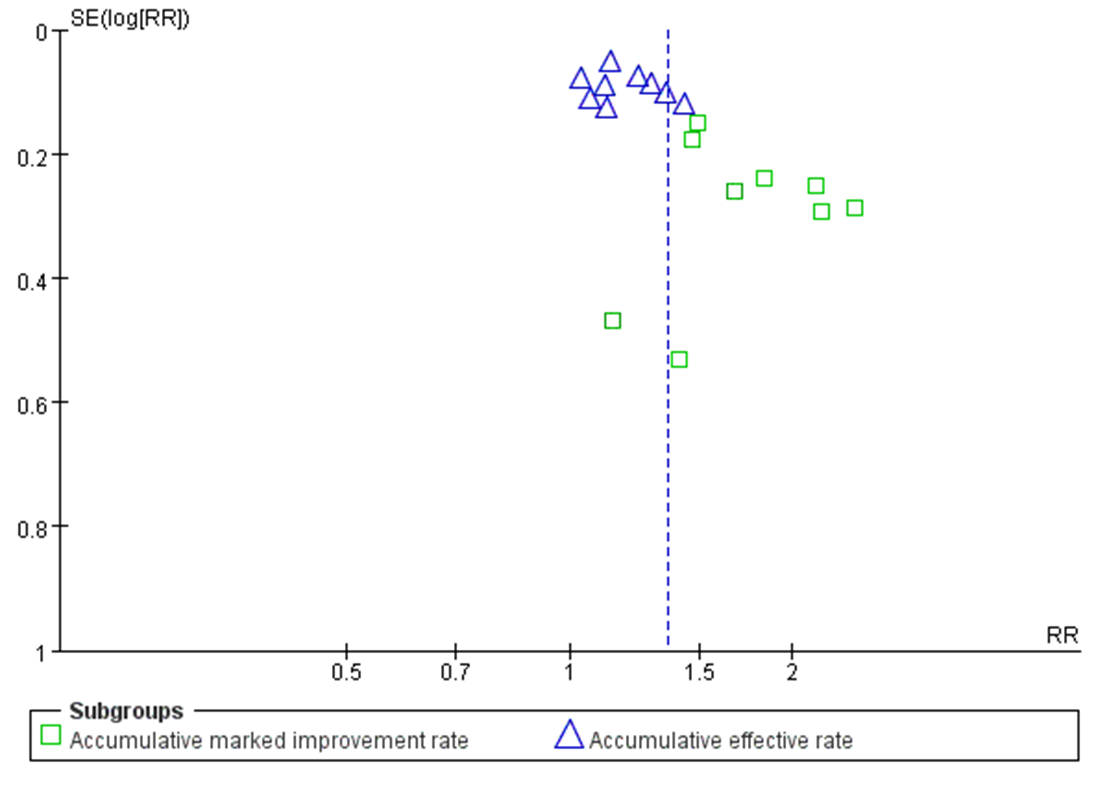
*

Figure S3: Guizhi Decoction associated formulas versus Western medicine


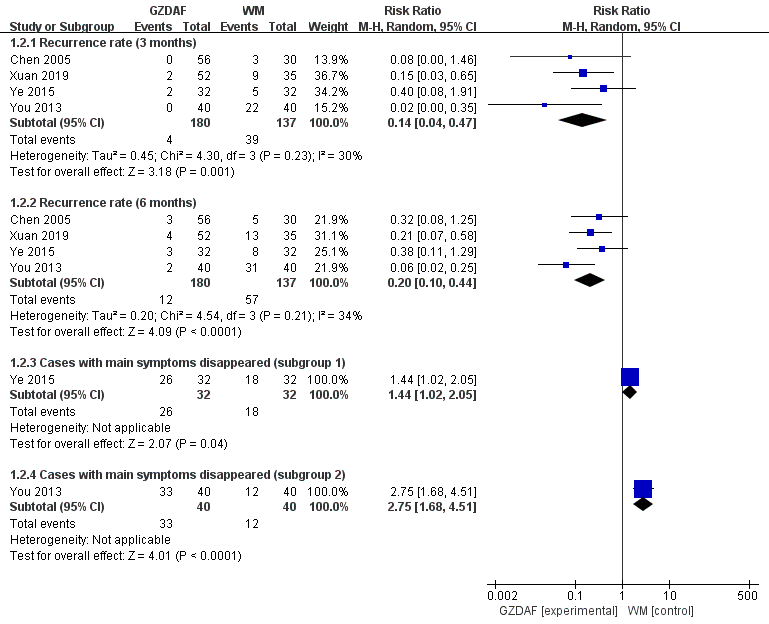


Figure S4: Guizhi Decoction associated formulas versus Western medicine-continued


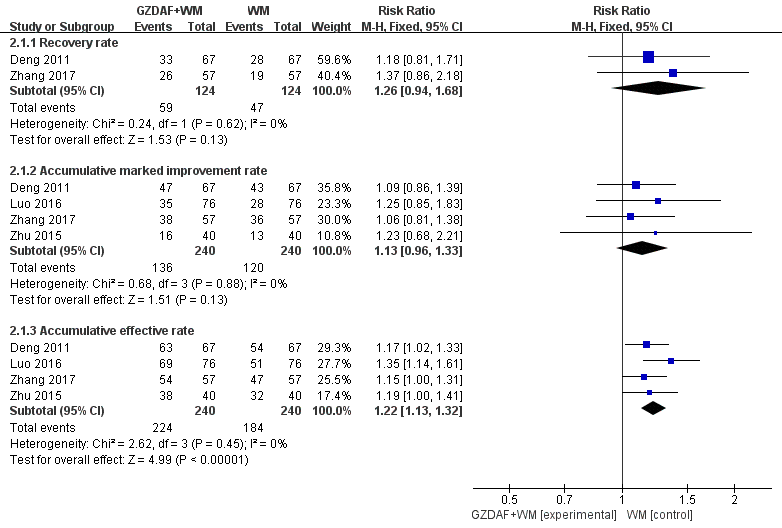


figure S5: Guizhi Decoction associated formulas plus Western medicine versus Western medicine


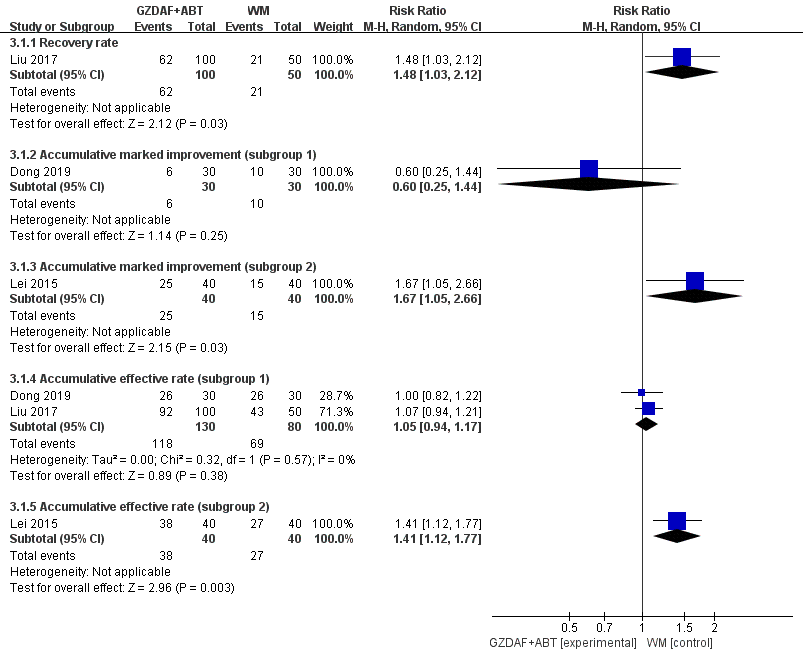


Figure S6: Guizhi Decoction associated formulas plus acupoint-based therapy versus Western medicine


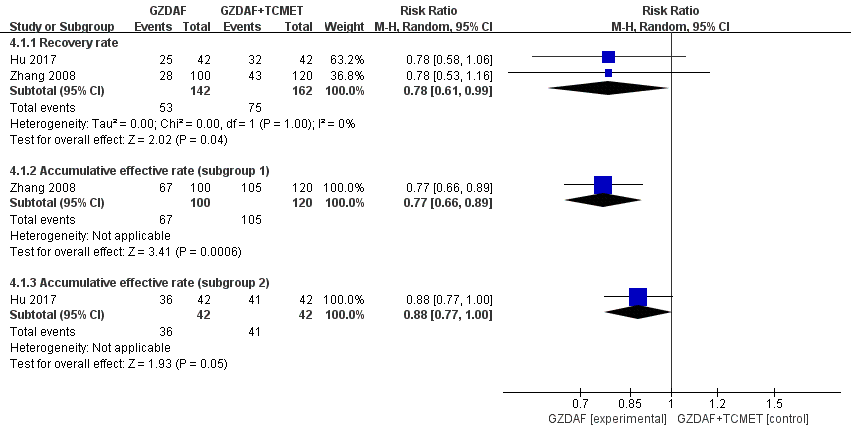


figure S7: Guizhi Decoction associated formulas versus Guizhi Decoction associated formulas plus TCM external therapy
